# Effect of Long COVID-19: Self-Reported Questionnaires and Objective Olfactory and Gustatory Evaluations

**DOI:** 10.3390/diseases14070256

**Published:** 2026-07-15

**Authors:** Ilenia Pinna, Paolo Solla, Gianni Orofino, Tommaso Ercoli, Francesco Salis, Francesco Loy, Carla Masala

**Affiliations:** 1 Department of Biomedical Sciences, University of Cagliari, SP 8 Cittadella Universitaria, 09042 Monserrato, Italy; f.salis27@studenti.unica.it (F.S.); floy@unica.it (F.L.); 2 Department of Neurology, University of Sassari, Viale S. Pietro 10, 07100 Sassari, Italy; psolla@uniss.it (P.S.); ercolitommaso@me.com (T.E.); 3 Department of Neurology, AOU Cagliari, University of Cagliari, SS 554 km 4.500, 09042 Cagliari, Italy; g.orofino@aoucagliari.it

**Keywords:** long COVID-19, olfactory dysfunction, gustatory dysfunction, chemosensory dysfunction

## Abstract

Background/Objectives: COVID-19 has triggered a global health crisis with sequelae extending beyond the acute phase of infection. Long COVID-19 is characterized by the persistence or new onset of symptoms weeks to months after the initial infection, including fatigue, cognitive impairment, and chemosensory dysfunction. Despite growing clinical evidence, the underlying pathophysiological mechanisms remain incompletely understood. This study aimed to evaluate the prevalence, distribution, and evolution of symptoms during the post-acute phases, with particular emphasis on olfactory and gustatory alterations. Methods: An observational study used a structured online questionnaire to collect demographic and clinical data from individuals with prior SARS-CoV-2 infection. Participants (*n* = 798) with persistent symptoms beyond 4–8 weeks were included. A subgroup of participants living in Sardinia underwent objective chemosensory assessment using the Sniffin’ Sticks test and the Taste Strips test for olfactory and gustatory function, respectively. Results: A high percentage of participants reported persistent symptoms consistent with long COVID-19, including fatigue, dyspnea, cognitive impairment, and chemosensory alterations. Olfactory and gustatory dysfunctions were among the most frequent manifestations. Objective assessments confirmed the presence and severity of these deficits, in accordance with data obtained using self-reported questionnaires. Symptom profiles evolved, suggesting possible central nervous system involvement and persistent inflammatory mechanisms. Conclusions: Long COVID-19 is a complex multisystem condition with a significant impact on quality of life. Chemosensory dysfunctions may serve as useful clinical markers for patient monitoring and stratification. Integrating subjective and objective data improves diagnostic accuracy and supports the development of targeted therapeutic strategies.

## 1. Introduction

COVID-19, caused by the SARS-CoV-2 virus, emerged in late 2019 in Wuhan, China, triggering a global pandemic with devastating effects on public health, healthcare systems, and the world economy [1]. Individuals infected with the SARS-CoV-2 virus typically develop symptoms 4 to 5 days after exposure [2]. Despite the introduction of effective vaccines and the development of antiviral therapies, COVID-19 continues to pose a significant threat. In addition to the acute phase of the disease, the phenomenon of long COVID-19 (LC) or post-COVID-19 syndrome has emerged in recent years [3,4,5,6]. This condition is characterized by persistent symptoms that may last for weeks or months following the initial infection. Long COVID-19 lacks a standardized definition but is generally characterized by the persistence of new or ongoing symptoms beyond 4 to 8 weeks after the acute phase of infection. These symptoms may include chronic fatigue, cognitive impairment (brain fog), myalgia, respiratory difficulties, and memory loss [7,8,9]. Although knowledge about the pathogenesis of COVID-19 has rapidly increased, long COVID-19 remains less understood. Long COVID-19 could be due to persistent immune activation, microthrombi formation, and reactivation of other viruses [10]. Long COVID-19 affects a significant proportion of the post-infection population, with estimates ranging from 10% to 20%, creating a substantial impact on public health and the quality of life of those affected [11]. The lack of a standardized diagnostic definition and specific tests complicates diagnosis and clinical management, highlighting the need for further research to improve the understanding of this condition [8,12]. COVID-19 infection and, subsequently, long COVID-19 have highlighted the crucial role of smell and taste in daily life [13]. During infection and subsequent phases, the olfactory and gustatory epithelia can undergo significant damage, leading to chemosensory dysfunction in many individuals [14,15,16,17]. Several studies have reported that patients with olfactory and gustatory dysfunctions have a higher predisposition to SARS-CoV-2 infection. Doty and colleagues found that patients with neurological deficits, such as anosmia and/or ageusia, are 17 times more likely to test positive for COVID-19 compared to those without these symptoms [14]. Although various hypotheses have been proposed to explain the mechanisms underlying the neurological symptoms of long COVID-19, the process of SARS-CoV-2 neuroinvasion remains unclear [18,19]. Ercoli and colleagues reported that approximately 35% of patients with acute COVID-19 infection experience persistent disturbances in smell and/or taste perception [7]. COVID-19-induced olfactory dysfunctions are frequently associated with structural and functional changes in brain areas involved in olfactory processing [19]. Several studies have assessed olfactory and gustatory function in the months following acute COVID-19 infection, using psychophysical tests, self-reported assessments, and resting-state functional MRI (rs-fMRI) [8,20]. Masala and colleagues highlighted that olfactory dysfunction is a significant chronic symptom in patients with long COVID-19 and is associated with central nervous system (CNS) involvement, as demonstrated using rs-fMRI [8]. This study aimed to evaluate a comprehensive analysis of the frequency and types of symptoms reported by patients in the acute phase of infection and the post-acute phase, commonly referred to as long COVID-19. The investigation aimed not only to assess the prevalence of symptoms across these phases but also to identify the possible emergence of new clinical manifestations or pathological conditions not previously present in post-acute COVID-19 syndrome (long COVID-19). The study examined a broad spectrum of clinical manifestations, including both symptoms classically associated with SARS-CoV-2 infection, such as fever, cough, fatigue, and dyspnea, and less common or more recently identified symptoms, such as olfactory and gustatory dysfunctions. Data collection and analysis enabled the identification of changes in symptom profiles over time, providing valuable insights into disease progression and its long-term impact on patients’ health. Particular attention was given to evaluating chemosensory symptoms reported by individuals with long COVID-19, a clinically complex and still-evolving condition characterized by the persistence of symptoms or the emergence of new sensory disturbances weeks or months after recovery from the acute phase of infection. As part of the investigation, participants in the online questionnaire were asked whether they lived in Sardinia, particularly near the University of Cagliari (Italy), and if they were willing to undergo objective testing for the assessment of chemosensory dysfunctions. This approach enabled the integration of self-reported symptom data with findings from standardized experimental assessments, thereby reducing the risk of bias associated with subjective symptom perception. Specifically, an objective analysis of olfactory and gustatory function was performed using specialized tests to determine with greater accuracy the presence and severity of potential chemosensory impairments. This methodological approach provided a more reliable evaluation of the impact of olfactory and gustatory dysfunctions in the context of long COVID-19. The integration of subjective and objective data contributed to a better understanding of the long-term sequelae of SARS-CoV-2 infection, offering valuable insights for the development of diagnostic and clinical management strategies for post-COVID sensory alterations.

## 2. Materials and Methods

### 2.1. Participants

In this study, 798 participants were included and stratified by sex (men and women) and age (≥25 years and <25 years). The age-based stratification was performed based on the median age, which is around 25 years.

The group labelled as men under 25 years of age (M < 25 years) included 86 participants (mean age 23.1 ± 1.5 years). The group labelled as men aged 25 years and older (M > 25 years) included 62 participants (mean age 32.1 ± 7.9 years). The group labelled women under 25 years (W < 25 years) included 429 participants (mean age 23.0 ± 1.6 years), while the group labelled women aged 25 or older (W > 25 years) included 221 participants (mean age 29.4 ± 5.9 years). The oldest participants were 59 and 63 years old for women and men, respectively.

This study was conducted using a telemedicine approach with an online questionnaire administered by Google Forms (Google LLC, Mountain View, CA, USA). The questionnaire was designed to collect detailed data on participants’ demographics, anamnesis, and symptoms, with a particular focus on their experiences with long COVID-19, including neurological and cardiac disorders. Additional data collected included any olfactory disturbances, whether directly related to SARS-CoV-2 infection or independent of it, as these symptoms have been frequently documented in the literature as indirect indicators of infection [7,8]. The questionnaire further explored the presence and types of symptoms experienced during the acute phase of the infection, as well as the persistence or emergence of new symptoms in the long COVID-19 phase. Particular attention was given to long-term symptoms, commonly known as “long COVID”, to identify possible correlations with neurological symptoms, fatigue, cognitive impairments, and other post-infection clinical manifestations. In addition to anamnesis and symptom-related questions, the Beck Depression Inventory-II (BDI-II), a validated tool for assessing depression [21], was administered. Data were collected using a nominal scale with responses coded as Yes = 1 and No = 0. This binary categorization allows for the classification of participants’ answers into two distinct groups. The use of a nominal scale ensures that the responses are treated as discrete, non-ordered categories, where each participant’s answer is classified as either “Yes” or “No” without any intrinsic hierarchy.

As mentioned in the introduction, a Sardinian subpopulation was evaluated at the University of Cagliari (Italy) from April 2023 to June 2023. A total of 48 participants were enrolled in the objective olfactory and gustatory tests with a mean age of 37.6 ± 13.1 years. Among them, 25 were women (mean age 33.7 ± 11.0 years), and 23 were men (mean age 41.7 ± 14.1 years).

In the patient group, the inclusion criteria were persistence of symptoms for at least 3 months following the initial SARS-CoV-2 infection. In the control group, only subjects who had never been affected by COVID-19 were enrolled. Additional inclusion criteria for the present study required that participants were adults aged 18 years or older.

For both patients and control groups, exclusion criteria included acute respiratory infections, neurodegenerative diseases, a history of head or neck trauma, chronic rhinitis or rhinosinusitis, asthma, stroke, diabetes, chronic renal disease, ongoing pregnancy, and any systemic condition associated with olfactory dysfunction.

The study was reviewed and approved by the ethics committee of the “Azienda Ospedaliero Universitaria di Cagliari” (PROT.NP/2023/963) and was performed in accordance with the Declaration of Helsinki. All experimental procedures were explained to participants, who signed the informed consent before the start of the experiment.

### 2.2. Subjective and Objective Clinical Evaluations

Participants’ olfactory function was assessed using the Sniffin’ Sticks test [22,23]. The Sniffin’ Sticks test consists of odor-filled pens to administer olfactory stimuli. During the test, the tip of the pen was positioned approximately 2 cm from the nostrils for about 3 s. Three parameters of olfactory function were evaluated: odor threshold (OT), odor discrimination (OD), and odor identification (OI) [24]. The OT was determined using n-butanol through a series of 16 stepwise dilutions. A single-staircase procedure based on a three-alternative forced-choice (3AFC) task was used for evaluation. The OD was assessed in 16 trials, each presenting three pens: two containing the same odor and one with a different (target) odor, using the 3AFC method. Finally, OI was tested using 16 common odors, each presented with four verbal descriptors, one correct and three distractors, in a multiple forced-choice format. An interval of 20–30 s was maintained between odor presentations. The total olfactory score (TDI) was calculated by summing the scores obtained in the three tests (OT, OD, and OI). These data indicate the potential presence of quantitative olfactory dysfunctions, which refer to alterations in odor intensity perception. The TDI score allows for the classification of olfactory function into four clinically relevant categories: anosmia (≤16), indicative of a complete or near-complete loss of smell; hyposmia (16.25–30.5), corresponding to a partial reduction in olfactory sensitivity; normosmia (30.75–41.25), representing an olfactory function within normal limits; and hyperosmia (>41.5), characterized by an above-average olfactory sensitivity [23,25]. Subsequently, the Taste Strips test (Burghart Messtechnik, Wedel, Germany) was used to evaluate gustatory function. This test involves filter paper strips impregnated with solutions at different concentrations for each of the four basic taste qualities: sweet (sucrose 0.4, 0.2, 0.1, 0.05 g/mL), bitter (quinine hydrochloride 0.006, 0.0024, 0.0009, 0.0004 g/mL), sour (citric acid 0.3, 0.165, 0.09, 0.05 g/mL), and salty (sodium chloride 0.25, 0.1, 0.04, 0.016 g/mL) [26]. Drinking water was provided both as a solvent for the taste solutions and to allow participants to rinse their mouths between tastings. The total gustatory score ranges from 0 to 16. A score of ≥9 indicates normogeusia, while a score of <9 is classified as hypogeusia [26].

### 2.3. Statistical Analyses

Statistical analyses for this study were conducted using GraphPad Prism 9.0.0 software (Boston, MA, USA). Based on previous studies using similar protocols [27,28]. About 798 total subjects could be considered suitable to detect statistical differences in the variables. Indeed, a power calculation was performed considering a critical effect size with a medium effect (f = 0.20–0.3) with a 95% power and 5% significance level in the Chi-square test (χ^2^). The demographic and clinical characteristics of the patients were indicated using mean ± standard deviation (SD) and percentages. Binary data indicated in percentages were evaluated using the chi-square test (χ^2^) to calculate statistical differences between different groups. A chi-square test (χ^2^) was performed to assess variations in olfactory function and the presence of comorbidities associated with concurrent conditions and long COVID-19. Additionally, Pearson’s bivariate correlation was performed to assess potential relationships between neurological symptoms before and during long COVID-19 infection. An unpaired *t*-test was calculated for olfactory and gustatory dysfunction during long COVID-19 in the Sardinian subpopulation compared to healthy controls. Statistical significance was defined as a *p*-value < 0.05.

## 3. Results

### 3.1. Demographic and Clinical Data of All Participants

Table 1 presents detailed clinical and demographic data of all participants, including age, sex distribution, and smoking status, as well as the prevalence of pre-existing medical conditions before the COVID-19 infection. Additionally, the table indicates the percentage of individuals who underwent molecular testing (RT-PCR) during symptomatic COVID-19 infection and the percentage of participants who had been vaccinated against COVID-19 before their infection. Furthermore, the table provides the average score of the BDI-II [21], a widely used measure for depressive symptoms assessment. These data collectively offer a comprehensive overview of the baseline characteristics and relevant clinical factors of the study participants.

Table 2 compares the symptoms during the acute COVID-19 infection and in long COVID-19. Patients during the acute COVID-19 infection showed significantly (*p* < 0.0001) higher flu symptoms compared to those obtained during long COVID-19 in all groups (M ≤ 25 years, M > 25 years, W ≤ 25 years, and W > 25 years). In men with ≤25 years, flu symptoms were found in 95.3% and 64.0% of patients for the COVID-19 and long COVID-19 groups, respectively (χ^2^ = 26.15, *p* < 0.0001). Similarly, in men with >25 years, flu symptoms were observed in 95.2% and 66.1% of patients with COVID-19 and long COVID-19, respectively (χ^2^ = 16.74, *p* < 0.0001). Instead, among women ≤ 25 years, flu symptoms were observed in 98.6% and 82.8% of patients in the COVID-19 and long COVID-19 groups (χ^2^ = 63.74, *p* < 0.0001), respectively. Also, in women with >25 years, flu symptoms were observed in 99.1% and 86.0% of patients in the COVID-19 and the long COVID-19 groups, respectively (χ^2^ = 27.54, *p* < 0.0001). In the long COVID-19 patients with <25 and >25 years, statistically significant differences between men and women were observed for flu symptoms (χ^2^ = 9.27, *p* < 0.01) and (χ^2^ = 10.96, *p* < 0.001), respectively.

The evaluation of olfactory dysfunction revealed that patients with COVID-19 exhibited a higher percentage compared to those with long COVID-19 in women with ≤25 years (χ^2^ = 56.72, *p* < 0.0001) and >25 years (χ^2^ = 11.52, *p* < 0.001), and only in men with >25 years (χ^2^ = 32.61, *p* < 0.0001). In the COVID-19 group, the percentages of patients with olfactory dysfunction were 24.2%, 20.5%, and 20.8% in the men with >25 years group, in women with ≤25 years, and >25 years group, respectively. While in the long COVID-19 group, the percentages of patients with olfactory dysfunction were 3.2%, 3.7%, and 3.2% in the men with >25 years group, and women with ≤25 years and >25 years, respectively. In men with <25 years, the percentages of olfactory dysfunction were 10.5% and 4.7% in the COVID-19 group and the long COVID-19 group, respectively. No statistically significant differences in olfactory dysfunction were observed between men and women in the COVID-19 and the long COVID-19 groups.

Gustatory dysfunction was significantly increased in women of both age groups (≤25 years and >25 years) in COVID-19 patients compared to those with long COVID-19. In detail, in women with ≤25 years, the percentage of patients with gustatory dysfunction was 21.2% and 7.9% in the COVID-19 and long COVID-19 groups (χ^2^ = 30.42, *p* < 0.0001), respectively. While in the group of women with >25 years, the percentage of patients with gustatory dysfunction was 19.5% and 9.0% in the COVID-19 and long COVID-19 groups (χ^2^ = 30.42, *p* < 0.01), respectively. No statistically significant differences for gustatory dysfunction were observed between men and women in the COVID-19 and the long COVID-19 groups.

Patients with acute COVID-19 infection reported higher levels of asthenia compared to those with long COVID-19, particularly in the following groups: M < 25 years, M > 25 years, W < 25 years, and W > 25 years. The M < 25 years group showed a statistically significant difference in asthenia symptoms, with 65.1% and 30.2% of patients in the COVID-19 group and long COVID-19 group (χ^2^ = 20.98, *p* < 0.0001), respectively. The M > 25 years group showed a statistically significant difference in asthenia symptoms, with 64.5% and 43.5% of patients in the COVID-19 group and in the long COVID-19 group (χ^2^= 5.49, *p* < 0.02), respectively. The W < 25 years group showed a statistically significant difference in asthenia symptoms, with 82.8% and 55.7% of patients in the COVID-19 group and in the long COVID-19 group (χ^2^ = 73.62, *p* < 0.0001), respectively. The W > 25 years group showed a statistically significant difference in asthenia, with 81.0% and 60.6% of patients in the COVID-19 group and the long COVID-19 group (χ^2^ = 22.17, *p* < 0.0001), respectively. In the COVID-19 and long COVID-19 patients with <25 and >25 years, statistically significant differences between men and women were observed for asthenia (χ^2^ = 8.42, *p* < 0.01), (χ^2^ = 13.79, *p* < 0.001), (χ^2^ = 6.49, *p* < 0.05), and (χ^2^ = 5.79, *p* < 0.05), respectively.

Furthermore, myalgia was reported by a high percentage of patients during the acute phase of COVID-19, in contrast to the long COVID-19 period, in the following groups: M < 25 years, M > 25 years, W < 25 years, and W > 25 years. The M < 25 years group showed a statistically significant difference in myalgia symptoms, with 52.3% and 7.0% of patients in the COVID-19 group and the long COVID-19 group (χ^2^= 42.39, *p* < 0.0001), respectively. The M > 25 years group showed a statistically significant difference in myalgia, with 56.5% and 11.3% of patients in the COVID-19 group and the long COVID-19 group (χ^2^ = 28.23, *p* < 0.0001), respectively. The W < 25 years group showed a statistically significant difference in myalgia symptoms, with 59.7% and 13.8% of patients in the COVID-19 group and the long COVID-19 group (χ^2^ = 197.7, *p* < 0.0001), respectively. The W > 25 years group showed a statistically significant difference in myalgia, with 66.1% and 18.6% of patients in the COVID-19 group and the long COVID-19 group (χ^2^ = 102.2, *p* < 0.0001), respectively. No statistically significant differences for myalgia were observed between men and women in the COVID-19 and the long COVID-19 groups.

Finally, data on the presence of dyspnea and cardiac conditions were analysed in patients during the acute phase of SARS-CoV-2 infection and in the subsequent period characterized by long COVID-19 syndrome. Statistical analysis revealed no statistically significant differences in the incidence of these conditions between the two phases, suggesting that cardiopulmonary symptoms may persist regardless of the clinical stage of the disease. Only for dyspnea, in the COVID-19 and long COVID-19 patients with <25 and >25 years, statistically significant differences between men and women were observed (χ^2^ = 7.45, *p* < 0.01), (χ^2^ = 8.86, *p* < 0.001), (χ^2^ =7.33, *p* < 0.01), and (χ^2^ = 4.37, *p* < 0.05), respectively. Instead, no statistically significant differences for cardiological pathologies were observed between men and women in the COVID-19 and the long COVID-19 groups.

**Figure 1:**
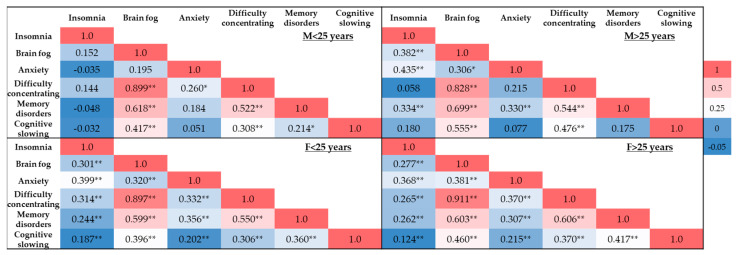
Heatmap of Pearson’s Correlation (*r*) between cognitive and neuropsychiatric symptoms by sex and age. Legend: Men under 25 years of age (M < 25 years), men over 25 years of age (M > 25 years), women under 25 years of age (W < 25 years), and women over 25 years of age (W > 25 years). * = *p* < 0.05, ** = *p* < 0.01.

Figure 1 shows correlation analyses with distinct patterns based on sex and age. Younger individuals exhibit more numerous and stronger associations, particularly between brain fog and difficulty concentrating, whereas older groups exhibit relationships but are less extensive. Overall, these findings indicate a strong interconnection between cognitive and psychological symptoms, with age and sex potentially acting as modulating factors. Brain fog emerges as the most central variable, showing consistently high positive correlations across all subgroups, especially with difficulty concentrating. Insomnia and anxiety also display significant associations, albeit more variable across groups, suggesting their contribution to the cognitive profile reported by participants. These results are in line with the literature, which identifies insomnia and anxiety as conditions associated with deficits in attention, memory, and overall cognitive performance, supporting the hypothesis of a bidirectional interaction among sleep disturbances, emotional state, and cognitive function [3,6,29].

In men under 25 years (M < 25), brain fog shows the highest positive correlation with difficulty concentrating (r = 0.899; *p* < 0.01), followed by memory disorders (r = 0.618; *p* < 0.01) and cognitive slowing (r = 0.417; *p* < 0.01). Anxiety is positively correlated with difficulty concentrating (r = 0.260; *p* < 0.05). In addition, difficulty concentrating is associated with both memory disorders (r = 0.522; *p* < 0.01) and cognitive slowing (r = 0.308; *p* < 0.01), while memory disorders correlate with cognitive slowing (r = 0.214; *p* < 0.05).

In men over 25 years (M > 25), insomnia shows positive correlations with brain fog (r = 0.382; *p* < 0.01), anxiety (r = 0.435; *p* < 0.01), and memory disorders (r = 0.334; *p* < 0.01). Brain fog is correlated with anxiety (r = 0.306; *p* < 0.05), difficulty concentrating (r = 0.828; *p* < 0.01), memory disorders (r = 0.699; *p* < 0.01), and cognitive slowing (r = 0.555; *p* < 0.01). Anxiety is associated with memory disorders (r = 0.330; *p* < 0.01), whereas difficulty concentrating correlates with both memory disorders (r = 0.544; *p* < 0.01) and cognitive slowing (r = 0.476; *p* < 0.01).

In women under 25 years (W < 25), insomnia correlates with all variables considered: brain fog (r = 0.301; *p* < 0.01), anxiety (r = 0.399; *p* < 0.01), difficulty concentrating (r = 0.314; *p* < 0.01), memory disorders (r = 0.244; *p* < 0.01), and cognitive slowing (r = 0.187; *p* < 0.01). Brain fog is significantly associated with anxiety (r = 0.320; *p* < 0.01), difficulty concentrating (r = 0.897; *p* < 0.01), memory disorders (r = 0.599; *p* < 0.01), and cognitive slowing (r = 0.396; *p* < 0.01). Anxiety correlates with difficulty concentrating (r = 0.332; *p* < 0.01), memory disorders (r = 0.356; *p* < 0.01), and cognitive slowing (r = 0.202; *p* < 0.01). Finally, difficulty concentrating is associated with memory disorders (r = 0.550; *p* < 0.01) and cognitive slowing (r = 0.306; *p* < 0.01), while memory disorders correlate with cognitive slowing (r = 0.360; *p* < 0.01).

In women over 25 years (W > 25), insomnia correlates with brain fog (r = 0.277; *p* < 0.01), anxiety (r = 0.368; *p* < 0.01), difficulty concentrating (r = 0.265; *p* < 0.01), memory disorders (r = 0.262; *p* < 0.01), and cognitive slowing (r = 0.124; *p* < 0.01). Brain fog shows significant correlations with anxiety (r = 0.381; *p* < 0.01), difficulty concentrating (r = 0.911; *p* < 0.01), memory disorders (r = 0.603; *p* < 0.01), and cognitive slowing (r = 0.460; *p* < 0.01). Anxiety is correlated with difficulty concentrating (r = 0.370; *p* < 0.01), memory disorders (r = 0.307; *p* < 0.01), and cognitive slowing (r = 0.215; *p* < 0.01). Difficulty concentrating is furthermore associated with memory disorders (r = 0.606; *p* < 0.01) and cognitive slowing (r = 0.370; *p* < 0.01), while memory disorders correlate with cognitive slowing (r = 0.417; *p* < 0.01).

### 3.2. Demographic and Clinical Data of the Sardinian Subpopulation

Table 3 shows the demographic characteristics of the Sardinian subpopulation of patients with long COVID-19 and healthy controls.

**Figure 2:**
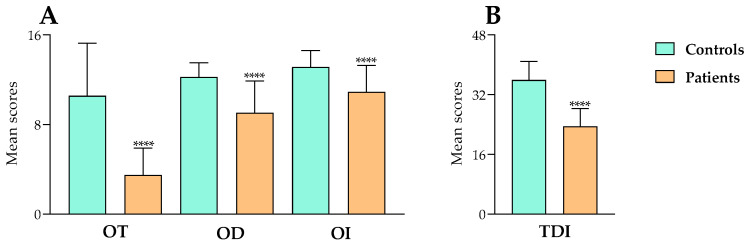
Comparison of mean olfactory test scores between the control and patients with long COVID-19 groups. (**A**) shows the odor threshold (OT), odor discrimination (OD), and odor identification (OI) scores, whereas (**B**) presents the total TDI score. Data are expressed as the mean ± standard deviation. **** = *p* < 0.0001. Statistical differences were calculated by unpaired *t*-test.

Figure 2A,B shows that patients with long COVID-19 exhibited significantly lower scores in olfactory function compared to controls. In Figure 2A, patients with long COVID-19 showed a significant decrease in mean OT scores compared to controls [t_94_ = 9.273, *p* < 0.0001, R^2^ = 0.477]. The mean OT scores were 3.5 ± 2.4 and 10.5 ± 4.8 in patients with long COVID-19 and in controls, respectively. Similarly, patients showed a significant decrease in OD mean score compared to controls [t_94_ =7.138, *p* < 0.001, R^2^ = 0.352]. The mean OD scores were 9.1 ± 2.8 and 12.3 ± 1.3, respectively, for patients and healthy controls. Moreover, patients showed a significant decrease in OI mean scores compared to controls [t_94_ =5.599, *p* < 0.0001, R^2^ = 0.250]. The OI mean scores were 10.9 ± 2.4 and 13.1 ± 1.5 for patients and controls, respectively. Consequently, in Figure 2B, patients exhibited a significant decrease in TDI score (which is the sum of OT, OD, and OI) compared to controls [t_94_ =12.520, *p* < 0.001, R^2^ = 0.625]. The mean TDI scores were 23.5 ± 4.8 and 35.8 ± 5.0 in patients and in healthy controls, respectively. Moreover, all patients with long COVID-19 exhibited quantitative olfactory dysfunction, with 91.7% and 8.3% presenting hyposmia and anosmia, respectively, whereas 10.4% of controls showed hyposmia.

**Figure 3:**
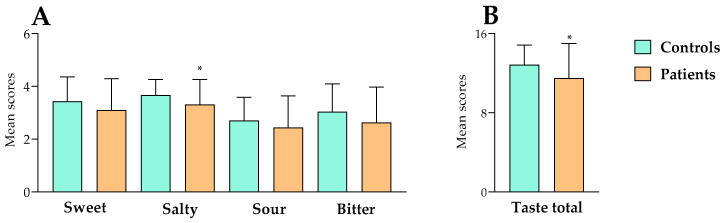
Comparison of mean gustatory test scores between control and patient groups with long COVID-19. (**A**) shows the comparison of mean scores for the sweet, salty, sour, and bitter taste qualities between control and patient groups, and (**B**) shows the total taste score. Data are expressed as the mean ± standard deviation. * = *p* < 0.05. Statistical differences were calculated by an unpaired *t*-test.

In the Taste Strip gustatory test (Figure 3A,B), patients with long COVID-19 exhibited significantly lower scores than controls, exclusively for the salty stimulus and in the total test score. Patients with long COVID-19 did not show a significant decrease in mean sweet scores compared to controls [t_94_ = 1.536, *p* > 0.05, R^2^ = 0.0245]. The mean sweet scores were 3.1 ± 1.2 and 3.4 ± 0.9 in patients and controls, respectively. On the other hand, patients with long COVID-19 showed a significant decrease in mean salty scores compared to controls [t_94_ =2.190, *p* < 0.05, R^2^ = 0.0486]. The mean salty scores were 3.3 ± 1.0 and 3.7 in patients and controls, respectively. Patients with long COVID-19 did not show a significant decrease in mean sour scores compared to controls [t_94_ =1.263, *p* > 0.05, R^2^ = 0.0167]. The mean sour scores were 2.4 ± 1.2 and 2.7 ± 0.9 in patients with and controls, respectively. Moreover, patients with long COVID-19 did not show a significant decrease in mean bitter scores compared to controls [t_94_ =1.690, *p* > 0.05, R^2^ = 0.0295]. The mean bitter scores were 2.6 ± 1.4 and 3.0 ± 1.1 in patients with and controls, respectively. Finally, in Figure 3B, patients with long COVID-19 showed a significant decrease in mean total taste scores compared to controls [t_94_ =2.330, *p* < 0.05, R^2^ = 0.055]. The mean total taste scores were 11.5 ± 3.5 and 12.9 ± 2.0 in patients with long COVID-19 and controls, respectively. Furthermore, 20.8% of patients with long COVID-19 exhibited hypogeusia, whereas 4.2% of healthy controls presented the same condition.

## 4. Discussion

The study utilized a mixed, integrated methodological approach, combining telemedicine with an anonymous online questionnaire administered via Google Forms to ensure maximum participant privacy, with objective chemosensory function tests [22,23,26] conducted at the laboratories of the University of Cagliari. This combined strategy represented an essential step to overcome the limitations of purely self-reported investigations, which are typical in many long COVID-19 studies [30], providing empirical validation of persistent symptoms. The primary objective was to comprehensively map the clinical and psychological symptoms in patients with long COVID-19, identifying symptomatic patterns useful for guiding future research initiatives and long-term care protocols. The questionnaire, developed by integrating validated scales from the literature with customized items on clinical history and persistent symptoms, assessed physical (e.g., asthenia and myalgia), sensory (olfactory and gustatory dysfunctions), and psychological (depression and anxiety) domains, enabling statistical analysis of disease burden [30]. Our results indicated a complex and prolonged symptomatic profile of SARS-CoV-2 infection, with particular emphasis on the chemosensory system, with olfactory and gustatory dysfunctions emerging as cardinal symptoms in the long COVID-19 phase. Our data are in line with the other previous studies [7,8,9,14,15,16,17,19,31,32].

These persistent deficits, often described as anosmia or hyposmia, likely reflect multiple pathogenic mechanisms, including direct viral neurotropism on the olfactory bulb, local inflammation, and post-viral immune dysregulation [16]. The persistent olfactory deficits could be due to the occurrence of severe damage and structural modifications in the olfactory epithelium and olfactory pathways, as previously reported using post-mortem histological analyses [33,34]. SARS-CoV-2 may enter the CNS using different pathways, such as direct passage of the virus across the axons of olfactory neurons or crossing the blood–brain barrier by the blood and the lymphatic systems [35].

Gustatory dysfunction (ageusia/hypogeusia) is significantly more prevalent in the acute phase among women cohorts (both ≤25 years and >25 years), hypothesizing greater gender-specific sensitivity in taste buds or a reporting bias related to somatic perception [36]. Conversely, olfactory dysfunction predominates in acute phases for women across all ages and men > 25 years, but not young men, suggesting gene-sex-specific interactions in olfactory pathways [36,37]. Although long-term impairments in smell and taste harm patients’ quality of life and do not receive adequate clinical attention.

Concurrently, mean scores on the BDI-II exceeded pre-pandemic normative cutoffs (mean ~10–12 in general populations), reaching values indicative of mild-to-moderate depressive symptomatology, consistent with other long COVID-19 studies conducted during and post-pandemic [8,21,30]. Emotions and olfactory function may share a common cerebral network, such as the amygdala, insula, hippocampus, anterior cingulate cortex, and orbitofrontal cortex [38].

Comparative analyses between acute and post-acute (long COVID-19) phases reveal distinct evolutionary dynamics: influenza-like symptoms (fever, acute myalgia, headache) universally dominate the initial phase, regardless of sex or age, consistent with the systemic inflammatory and cytokine response typical of viral replication [39].

Persistent symptoms such as asthenia and myalgia, though reduced in intensity, affect a substantial proportion of patients in long COVID-19, plausibly sustained by residual muscle inflammation, microcoagulopathies, or neuro-immune dysregulation, as hypothesized in recent models [29,40,41,42].

An original contribution of the study arises from the correlation analysis between cognitive and psychological domains, revealing a highly interconnected network where brain fog, concentration difficulties, memory disturbances, and cognitive slowing act as central nodes, particularly intense in younger subjects (≤25 years) [10]. The strongest association—between brain fog and concentration difficulties—supports the conceptualization of brain fog not as a vague symptom, but as an executive-attentional syndrome dominated by deficits in focus, slow processing, and reduced cognitive efficiency, with functional impacts on complex daily activities (e.g., work and study) [19]. These correlations extend to mnestic disturbances and slowing, suggesting a shared substrate of prefrontal and attentional inefficiency rather than isolated deficits [17]. Insomnia and anxiety emerge as key modulators: insomnia positively correlates with all cognitive parameters (brain fog, concentration, memory, slowing) consistently across groups, aligning with experimental evidence linking sleep deprivation to reduced synaptic plasticity, working memory deficits, and perceptual clouding [10].

Similarly, anxiety—via amygdala-cortical hyperactivation and elevated cortisol—saturates attentional resources, exacerbating brain fog and interfering with hippocampal consolidation [10,19]. In older groups (>25 years), networks are less dense but significant, attributable to accumulated cognitive reserve, emotional regulatory maturity, and compensatory strategies, despite persistent sleep-cognition links [8]. These patterns bolster multifactorial models of brain fog in long COVID-19, proposing bidirectional vicious cycles (e.g., anxiety → insomnia → cognitive deficits → amplified distress) and opportunities for integrated interventions (sleep hygiene, CBT, mindfulness).

Moreover, an important observation emerged among women of childbearing age, where 14.30% reported menstrual cycle alterations (e.g., amenorrhea, irregularity, or dysmenorrhea) following recovery from acute infection [30]. Although such evidence is preliminary and subject to intrinsic limitations of the self-reported design, such as recall bias and psychosocial confounding, it aligns with anecdotal reports and observational studies suggesting an indirect impact of the pandemic (chronic stress, lockdown, systemic inflammation) on the hypothalamic-pituitary-ovarian axis [21,30]. Interpretative caution is recommended, with a need for prospective investigations incorporating hormonal assays and controls for confounding variables. The absence of repeated longitudinal follow-ups, dictated by the questionnaire’s anonymous, one-shot design, constitutes a limitation, yet the data underscore a persistent psychological burden, potentially linked to neurogenic inflammation and altered limbic circuits [8].

In the Sardinian subpopulation with long COVID-19, the inclusion of objective olfactory and gustatory function tests showed statistical differences in olfactory function and gustatory tests between patients with long COVID-19 and their age-matched healthy controls. In patients with long COVID-19, a significant decrease was observed in all parameters of olfactory function, such as threshold, discrimination, and identification. However, in long COVID-19 patients, we observed a smaller reduction in gustatory function that was linked only to the saltiness sensory modality. A high prevalence of olfactory dysfunction in patients with long COVID-19 and a less frequent impairment in taste qualities has been previously reported [31,35]. The impairment in olfactory function may be mediated by direct action of SARS-CoV-2 on olfactory receptors, with an inflammatory reaction mediated by interleukin-6 and disruption of the olfactory epithelium [33].

As regards gustatory function, the Taste Strips test revealed selective deficits for salty stimulus and total score, without significant differences for sweet, bitter, or sour, defining a salt-specific hypogeusia. The salt-specific hypogeusia profile potentially reflects the selective vulnerability of salty taste buds or alterations in chorda tympani nerve pathways [43,44,45]. The salty taste plays a crucial role in osmotic balance, water homeostasis, and neuronal activity.

However, our data are in line with a previous Italian study [46] and differ from those reported in Asadi’s study since they indicate a decrease in the perception threshold for salty taste. This difference is due to the use of self-reported tests, whereas we employ an objective assessment of gustatory function.

In addition, SARS-CoV-2 may bind to sialic acid receptors in the taste buds and may induce the degradation of the gustatory system [47]. SARS-CoV-2 has competitive activity on ACE2 receptors in the taste buds [48]. Sialic acid plays a fundamental role in the salivary mucin, and its reduction is related to an increase in the gustatory threshold [47]. Another possibility could be the concomitant presence of olfactory deficits in patients with hypogeusia, due to the high relationship between olfactory and gustatory networks. Gustatory dysfunction could be related to the destruction of the papillae or taste buds, upper airway infection, poor oral health, and post-viral cranial. The destruction of the papillae or taste buds could be increased by changes in sodium homeostasis and saliva composition in COVID-19 infection.

This study shows the following limitations: the absence of longitudinal assessments in individual participants precludes analysis of temporal evolution; the anonymous online design introduces selection bias (digitally savvy and motivated participants) and recall bias; the regional sample (Sardinia, Cagliari-centric) limits generalizability, potentially influenced by local genetic factors (e.g., Sardinian gene pool) or viral variants; confounding variables such as vaccination status, comorbidities, or pharmacotherapy were not deeply extracted [8]. The absence of repeated longitudinal follow-ups, dictated by the questionnaire’s anonymous, one-shot design, constitutes a limitation, yet the data underscore a persistent psychological burden, potentially linked to neurogenic inflammation and altered limbic circuits [8]. The significant sex imbalance in participation is primarily attributable to men’s lower propensity to complete questionnaires, a phenomenon well documented in epidemiological research. Men are less likely to respond to surveys, particularly in relation to health-related and psychosocial topics. This behavior has been linked to reduced health-seeking attitudes and a lower tendency to report subjective symptoms. Consequently, the observed imbalance likely reflects a participation bias rather than a true sex-specific difference in the prevalence of olfactory and gustatory dysfunction.

Future studies are necessary to evaluate prospective multicentric designs, including biomarkers (such as cytokines, neuroimaging, tissue viral PCR), genotyping, and extended follow-ups.

## 5. Conclusions

In summary, our study highlights the multisystemic and persistent impact of long COVID-19 across sensory, cognitive, and psychological domains, validating the utility of mixed methods (self-report questionnaires and objective tests) to bridge subjective-objective discrepancies and delineate the true burden. Phase-specific patterns, age- and sex-modulated cognitive-psychological networks, and selective chemosensory deficits provide valuable insights for patient stratification and therapeutic trial design. Clinically, routine integration of olfactory-gustatory tests and cognitive screenings into post-COVID protocols is recommended, with an emphasis on personalized rehabilitation (olfactory training and sleep-anxiety management), as previously indicated [10]. Scientifically, these data, the first extensive Sardinian study on long COVID-19, lay the groundwork for further investigations and interventional studies, helping to fill gaps in the literature. Further research that encompasses diverse populations and uses longitudinal designs is imperative to translate these findings into effective, predictive therapeutic strategies.

## Figures and Tables

**Table 1:** Clinical and demographic information of all participants in this study is divided by gender and age. Data are expressed in percentages or mean ± SD.

	Total	M ≤ 25 Years	M > 25 Years	W ≤ 25 Years	W > 25 Years
Participants	798	86	62	429	221
Age	25.5 ± 5.3	23.1 ± 1.5	32.1 ± 7.9	23.0 ± 1.6	29.4 ± 5.9
Smoking status (% smokers)	32.3%	33.7%	41.9%	33.3%	27.1%
Presence of pathologies before COVID-19 infection (%)	20.0%	10.5%	8.1%	20.7%	25.8%
Average duration of COVID-19 positivity	9.9 ± 14.7	10.1 ± 20.7	10.1 ± 13.2	9.8 ± 15.3	10.0 ± 10.8
Swab type (% molecular swab)	31.7%	30.2%	35.5%	28.4%	37.6%
Vaccination against COVID-19 before positivity	79.1%	76.7%	79.0%	79.7%	78.7%
Depression (BDI-II)	12.0 ± 9.8	10.4 ± 10.3	8.9 ± 9.2	13.1 ± 9.8	11.3 ± 9.6

Legend: BDI-II = Beck Depression Inventory-II; Men under 25 years of age (M < 25 years), men over 25 years of age (M > 25 years), women under 25 years of age (W < 25 years), and women over 25 years of age (W > 25 years). SD = standard deviation.

**Table 2:** Comparison between patients with COVID-19 and long COVID-19 symptoms in relation to sex and age.

Percentages					Significance			
	M < 25 Years	M > 25 Years	W < 25 Years	W > 25 Years	M < 25 years	M > 25 Years	W < 25 Years	W > 25 Years
					C/LC	C/LC	C/LC	C/LC
Flu symptoms								
C	95.3%	95.2%	98.6%	99.1%	***p*** **< 0.0001**	***p*** **< 0.0001**	***p*** **< 0.0001**	***p*** **< 0.0001**
LC	64.0%	66.1%	82.8%	86.0%				
Olfactory dysfunction								
C	10.5%	24.2%	20.5%	20.8%	*p* > 0.05	***p*** **< 0.0001**	***p*** **< 0.0001**	***p*** **< 0.0001**
LC	4.7%	3.2%	3.7%	3.2%				
Gustatory dysfunction								
C	11.6%	22.6%	21.2%	19.5%	*p* > 0.05	*p* > 0.05	***p*** **< 0.0001**	***p*** **< 0.01**
LC	10.5%	9.7%	7.9%	9.0%				
Asthenia								
C	65.1%	64.5%	82.8%	81.0%	***p*** **< 0.0001**	***p*** **< 0.02**	***p*** **< 0.0001**	***p*** **< 0.0001**
LC	30.2%	43.5%	55.7%	60.6%				
Myalgia								
C	52.3%	56.5%	59.7%	66.1%	***p*** **< 0.0001**	***p*** **< 0.0001**	***p*** **< 0.0001**	***p*** **< 0.0001**
LC	7.0%	11.3%	13.8%	18.6%				
Dyspnoea								
C	23.3%	24.2%	40.6%	41.6%	*p* > 0.05	*p* > 0.05	*p* > 0.05	*p* > 0.05
LC	18.6%	27.4%	38.2%	40.7%				
Cardiological pathologies								
C	4.7%	6.5%	5.8%	4.5%	*p* > 0.05	*p* > 0.05	*p* > 0.05	*p* > 0.05
LC	1.2%	1.6%	5.6%	7.2%				

Legend: Symptoms experienced during COVID-19 (C); symptoms experienced during Long COVID-19 (LC). Men participants under 25 years of age (M < 25 years), men over 25 years of age (M > 25 years), women under 25 years of age (W < 25 years), and women over 25 years of age (W > 25 years). Bold indicates statistically significant data calculated using chi-square test (χ^2^).

**Table 3:** Demographic and chemosensory information for the Sardinian subpopulation divided between patients and controls. Data are expressed as the mean ± standard deviation (SD).

	Patients	Controls	*p* Value
Participants	48	48	*p* > 0.05
Sex N (% women)	52.1%	56.3%	*p* > 0.05
Age	37.6 ± 13.1	37.5 ± 12.0	*p* > 0.05
Smoking status (%smoker)	66.7%	77.1%	*p* > 0.05

Legend: N = number.

## Data Availability

The data presented in this study are available on request from the corresponding author. (The data are not publicly available due to ethical restrictions.)

## Abbreviations

The following abbreviations are used in this manuscript:

3AFC	Three-alternative forced-choice
ACE2	Angiotensin-converting enzyme 2
BDI-II	Beck depression inventory-II
C	Symptoms experienced during COVID-19
CNS	Central nervous system
LC	Symptoms experienced during long COVID-19
M < 25 years	Men under 25 years of age
M > 25 years	Men over 25 years of age
OD	Odor discrimination
OI	Odor identification
OT	Odor threshold
SD	Standard deviation
TDI	Total olfactory score
W < 25 years	Women under 25 years of age
W > 25 years	Women over 25 years of age
